# Notice

**Published:** 1884-09

**Authors:** William Carr

**Affiliations:** 35 West 46th Street, N. Y., Chairman of the Committee


### NOTICE.

The Committee on Enforcement of the New York State Dental Law desire that the name of every one who attempts the practice of dentistry illegally shall be reported to them. Every dentist who is not duly registered in the county clerk's office of his county, or who is illegally registered, is liable to the penalty provided in the act. If he shall have commenced practice since June 20, 1879, he must register his credentials—either the diploma of the Dental Society of the State of New York, or that of some reputable college, *recognized as such by that Society.* WILLIAM CARR,

35 West 46th Street, N. Y., Chairman of the Committee.

